# A Statistical Approach to Correcting Cross-Annotations in a Metagenomic Functional Profile Generated by Short Reads

**DOI:** 10.4172/2155-6180.1000208

**Published:** 2014-11-10

**Authors:** Ruofei Du, Donald Mercante, Lingling An, Zhide Fang

**Affiliations:** 1 Biostatistics Program, School of Public Health, Louisiana State University Health Sciences Center, New Orleans, Louisiana, USA; 2 Department of Agricultural and Bio-systems Engineering, University of Arizona, Tucson, Arizona, USA

**Keywords:** Metagenome, Functional profiling, Short reads, Probabilistic latent semantic analysis

## Abstract

**Background:**

Categorizing protein coding sequences into one family, if the proteins they encode perform the same biochemical function, and then tabulating the relative abundances among all the families, is a widely-adopted practice for functional profiling of a metagenomic sample. By homology searching of metagenomic sequencing reads against a protein database, the relative abundance of a family can be represented by the number of reads aligned to its members. However, it has been observed that, for short reads generated by next-generation sequencing platforms, some may be erroneously assigned to the functional families they are not associated to. This commonly occurred phenomenon is termed as cross-annotation. Current methods for functional profiling of a metagenomic sample use empirical cutoff values, to select the alignments and ignore such cross-annotation problem, or employ summarized equation to do a simple adjustment.

**Result:**

By introducing latent variables, we use the Probabilistic Latent Semantic Analysis to model the proportions of reads assigned to functional families in a metagenomic sample. The approach can be applied on a metagenomic sample after the list of the true functional families being obtained or estimated. It was implemented in metagenomic samples functionally characterized by the database of Clusters of Orthologous Groups of proteins, and successfully addressed the cross-annotation issue on both *in vitro*-simulated, bioinformatics tool simulated metagenomic samples, and a real-world data.

**Conclusions:**

Correcting cross-annotation will increase the accuracy of the functional profiling of a metagenome generated by short reads. It will further benefit differential abundance analysis of metagenomic samples under different conditions.

## Background

Microbiota plays an important role, beneficial or harmful, in many aspects of environment and our daily life. The study of microbial genetic material obtained directly from environmental/clinical samples, the so called metagenomics, has become a widely-used methodology to learn about a microbial community [1]. Aiming to characterize microbial communities residing in natural ecosystems or biologically host associated systems, metagenomic samples have been taken from various kinds of environments: seawater [2], soil [3], mine drainage [4], human or animal’s oral cavity [5,6], gut system [7,8], and so on. One of the major interests from collecting these samples is to reveal the diversity and abundance of biochemical functions associated to a microbial community [9].

Protein coding sequences (CDSs) contained in genomes can indicate the potential for a microbial community to encode proteins, which link to different biochemical functions in cells. Categorizing CDSs into one family if the proteins they encode perform the same function, and tabulating the relative abundances among all the families, is a widely adopted practice for functional profiling of a metagenomic sample [10]. Specifically, in a metagenomic study, the sequencing reads are translated to all possible reading frames and then aligned against a protein/domain sequence database, for example, the Clusters of Orthologous Groups of proteins (COG) [11,12] or Eukaryotic Orthologous Groups of proteins (KOG) [13], the collections of protein families PFAM [14] and TIGRFAMs [15], such that a read can be assigned to a protein functional family. The list of all the detected functional families and the corresponding proportions of counts of the reads to these families present the functional profile of the metagenomic sample. This is the so called read-count approach [16].

The next-generation sequencing (NGS) technologies such as Roche’s 454 Life Sciences, Illumina/Solexa, and Applied Biosystems’ SOLiD adopt an array-based work flow, which is exponentially faster than traditional chain-termination methods. These technologies do not require DNA cloning, and thus can avoid the cloning bias associated with the traditional Sanger sequencing technology [17]. Meanwhile, the sequencing cost has been dramatically reduced. These advantages have made the NGS technologies more and more preferred. Currently, one can hardly find a metagenomic project which does not choose a NGS technology.

Compared to Sanger sequencing, NGS technologies produce relatively short reads. Some NGS platforms produce sequencing reads with average length about 100 bases. However, it has been shown that the mean length of CDSs is highly conserved in prokaryotes, and is estimated to be about 924 base pairs [18]. Thus, when a translated short read is aligned to a protein/domain sequence, the alignment actually finds the sequence similarity between the translated read and a fragment of the protein/domain sequence. This may affect the alignment accuracy. First, it may violate an assumption for BLAST [19,20] to compute the significance of sequence similarity, which requires that the lengths of two sequences compared are sufficiently long [21]. Researchers had to use different “conventional” or “empirical” cutoff values to choose the alignments with significant sequence similarity, for example, BLAST E-value cutoffs 10^−3^, 10^−5^ [7,9,22]. It has been observed that, a large part of homologues, which can be detected by BLAST searching with long reads, are missed by searching with short reads using these E-value cutoffs [22]. In our recent paper [23], we proposed taking a number between 63 and 68 (default as 66) of BLAST similarity score as the cutoff to choose homologues, when aligning short reads with ~100 bases against COG database. We further suggested, through comparing the alignment output by RPS-BLAST on the same sample, to estimate artificial COGs in the BLAST output after cutoff.

**Figure 1:**
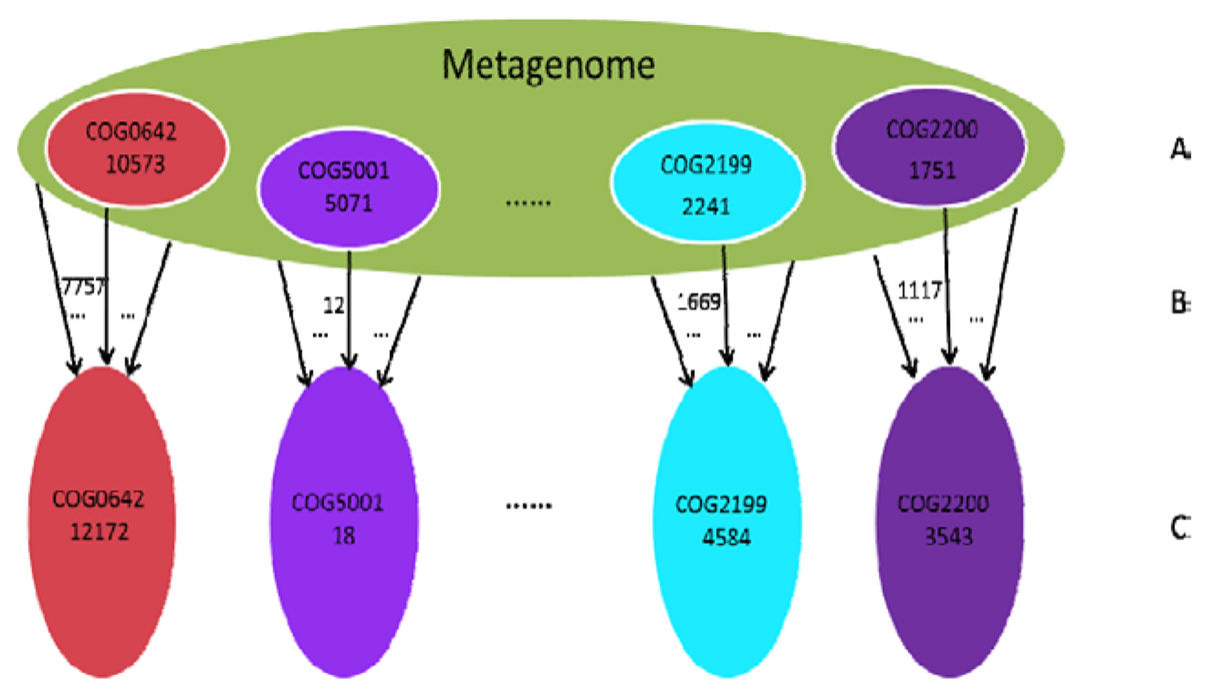
An example of cross-annotation in the aligned reads. (A) The true number of reads to each of the four COG families in the metagenome. (B) For each COG, only a partial number of the true reads being correctly assigned to the same COG. (C) In the alignment output, the read count to a COG includes the number of correctly assigned reads (from the same COG), and the number of erroneously assigned reads (from other COGs).

Zhang et al. [16] pointed out another issue in the read-count approach with short reads, that is, different functional families tend to have different proportions of wrong annotations. We observed the same problem when analyzing the *in vitro*-Simulated data set M_4X (details later). For example, in the BLAST searching output after filtration by the score cutoff 66, the counts of reads assigned to COG0642, COG5001, COG2199, and COG2200 are 12172, 18, 4584 and 3543 respectively. But, only partial numbers of these reads truly originate from the CDSs to which they are associated. There are 7757, 12, 1669 and 1117 such reads correspondingly. This indicates that a non-negligible proportion of aligned reads, for example 4415 (12172 minus 7757) reads to COG0642, are actually associated to other COGs. Meanwhile, we know that the true counts of reads to these four COGs should be 10573, 5071, 2241, and 1751 respectively. This implies that many reads from a COG, for example 2816 (10573 minus 7757) reads from COG0642, can be erroneously assigned to other COG families. These phenomena together define cross-annotation and are demonstrated in Figure 1.

The above examples show that the problem of cross-annotation is not trivial and will greatly affect the accuracy of the functional profiling if not being addressed properly. In this paper, we propose a method to mitigate the cross-annotation effect and improve the accuracy of estimates of read counts assigned to the functional families.

## Methods

In construction of functional profile of a metogenomic sample by the read-count approach, given the total number of reads and the probability that a read is generated from a COG family, the expected count of reads originated from the family can be easily calculated following a multinomial rule. Thus, accurately estimating the probability that a read is generated from a COG can certainly reduce the cross-annotation effect. We apply Probabilistic Latent Semantic Analysis (PLSA, details next) to estimate these probabilities, and then the proportions of reads originated from the estimated existing COGs.

### Input data

The metagenomic short reads with about 100 bases are BLAST (specifically, blastx) aligned against the COG database. A read is assigned to a COG family according to its best-hit association. The raw functional profile, consisting of the list of all detected COGs and corresponding relative abundances (quantified by the counts of reads assigned), may include artificial COGs and have the cross-annotation issue. Following the work-flow in Du et al., [23], the BLAST alignments with similarity score greater than 66 are retained and the artificial COGs are identified and removed. Furthermore, we treat a COG family as an artifact as well, if it has zero read count in the RPS-BLAST output after filtration by the similarity score 61. Then the input data for PLSA modeling consist of the following parts:

1. The COGs, to which the sequencing reads have been aligned;
2. The count of reads assigned to each COG in (1);
3. The estimated existing COGs (that is, the non-artificial COGs), and one extra family which covers the CDSs that exist but are not classified into COG families, and all the existing non-coding sequences in the sample.

### PLSA modeling

PLSA is a statistical modeling technique originally developed for information retrieval from text collections [24]. In the following, we will show how PLSA modeling is used to correct the cross-annotations. Suppose that the metagenomic sequencing reads are aligned to N different COG families, of which there are M truly existing COGs (*M* ≤ *N*). Define

*A*: one of the *N* COGs, denoted by *a_1_, a_2_*… *a_N_*, to which a read is aligned;
*T*: one of the *M* COGs, denoted by *c_1_, c_2_*… *c_M_,* from which a read originates;
*α_ij_*: the probability of a read being aligned to *a_i_* given that it originates from *c_j_*, that is, *α_ij_=P(A=a_i_ |T =c_j_)* or *P(a_i_|c_j_)*;
*β_j_*: the probability of an aligned read being from the COG *c_j_* in the metagenomic sample, that is, *β_j=_P(T=c_j_)* or *P(c_j_)*;
*y_i_* : the observed count of reads being aligned to the COG *a_i_*;
$t_{ir}^{u}$: The unobserved value of *T* for the *r^th^* read aligned to COG *a_i_*, where *r=1,*…*, y_i_*.

Then, the probability of a read originating from *c_j_* and being aligned to *a_i_* is

$$P\;(A=a_{i},T=c_{j})=P\;(A=a_{i}|T=c_{j})P\;(T=c_{j})=\alpha_{ij}\;\beta_{j}.$$

However, it is unobservable which COG an aligned read originates from. Thus, for any COG *a_i_* and the corresponding count *y_i_*, the probability that the *r^th^* read (*r=1,*…*, y_i_*) is from one of the M COGs, *c_1_, c_2_* … *c_M_*, and aligned to *a_i_* can be written as:

$$\sum_{j=1}^{M}I(t_{ir}^{u}=c_{j})P(A=a_{i},T=c_{j})=\sum_{j=1}^{M}I_{ir}(c_{j})\alpha_{ij}\beta_{j}.$$

Note that this sum has only one non-zero term because a read originates from only one COG.

For any *i* ∈ {*1,2,*…*, N*}, under the assumption that a read being aligned to COG *a_i_* is independent of another read being aligned to *a_i_*, we have the following likelihood function of (*α_i1_, α_i2_,*…*, α_iM_, β_1_, β_2_,*…*, β_M_*):

$$L(\alpha_{i1},\alpha_{i2},\cdots \alpha_{iM},\beta_{1},\beta_{2}\cdots \beta_{M}|y_{i},t_{i1}^{u},t_{i2}^{u},\cdots t_{iy_{i}}^{u})=\prod_{r=1}^{y_{i}}\sum_{j=1}^{M}I_{ir}(c_{j})\alpha_{ij}\beta_{j}.$$

Further assume that a read being aligned to COG *a_i_* is independent of the read being aligned to another COG, then the likelihood function and the log-likelihood function of *(α, β)* are

$$L(\alpha ,\beta |y,t^{u})=\prod_{i=1}^{N}\prod_{r=1}^{y_{i}}\sum_{j=1}^{M}I_{ir}(c_{j})\alpha_{ij}\beta_{j},$$

$$l(\alpha ,\beta |y,t^{u})=\log L(\alpha ,\beta |y,t^{u})=\sum_{i=1}^{N}\sum_{r=1}^{y_{i}}\log\left(\sum_{j=1}^{M}I_{ir}(c_{j})\alpha_{ij}\beta_{j}\right)\;=\sum_{i=1}^{N}\sum_{r=1}^{y_{i}}\sum_{j=1}^{M}I_{ir}(c_{j})\log(\alpha_{ij}\beta_{j}),$$

where α denotes the vector (*α*_11_, *α*_12_, ⋯, *α*_1_*_M_*, *α*_21_, *α*_22_, ⋯, *α*_2_*_M_*, ⋯, *α_N_*_1_, *α_N_*_2_, ⋯, *α_NM_*)′; β denotes the vector (*β*_1_, *β*_2_, ⋯, *β_M_*)′; y denotes the vector (*y*_1_, y_2_, ⋯, y*_N_*)′; and t^u^ denotes the vector ${\left(t_{11}^{u},t_{12}^{u},.....t_{1y_{1}}^{u},t_{21}^{u},t_{22}^{u},......t_{2y_{2}}^{u},.....,t_{N2}^{u},\ldots t_{Ny_{N}}^{u}\right)}^{\prime }$.

Given the observed counts {y_i_}, our goal is to find the estimates (Maximum Likelihood Estimates, MLEs) of parameters α, β. However this could not be done by maximizing the likelihood directly since t^u^, the realization of T, is unobservable. Nevertheless, by treating T as a latent variable, we can apply the Expectation-Maximization (EM) algorithm to search for the MLEs of α, β. Next, we describe in detail the iteration steps of the algorithm, but postpone the setting of the initial values to Section 4.3.

#### E step

In this step, we calculate the expected value of the log-likelihood function with respect to the condition distribution of T |y, θ^(k)^, where θ^(k)^ stands for the current estimate of θ=(α,β)′. By Bayes’ rule, for a read being aligned to COG *a_i_*, the conditional probability that it is from COG *c_j_* is

$$P\left(T=c_{j}|y,\theta^{\left(k\right)}\right)=\frac{\alpha_{ij}^{\left(k\right)}\beta_{j}^{\left(k\right)}}{\sum_{s=1}^{M}\alpha_{is}^{\left(k\right)}\beta_{s}^{\left(k\right)}}.$$

The expectation of the log-likelihood is

$$Q\left(\theta |\theta^{\left(k\right)}\right)=E_{T|Y,\theta^{\left(k\right)}}\left(l\left(\theta |y,T\right)\right)\;=\sum_{i=1}^{N}\sum_{r=1}^{y_{i}}\sum_{j=1}^{M}E_{T|Y,\theta^{\left(k\right)}}\left(I_{ir}\left(c_{j}\right)\log\left(\alpha_{ij}\beta_{j}\right)\right)\;=\sum_{i=1}^{N}\sum_{j=1}^{M}y_{i}\frac{\alpha_{ij}^{\left(k\right)}\beta_{j}^{\left(k\right)}}{\sum_{s=1}^{M}\alpha_{is}^{\left(k\right)}\beta_{s}^{\left(k\right)}}\log\left(\alpha_{ij}\beta_{j}\right)\;=\sum_{i=1}^{N}\sum_{j=1}^{M}y_{i}\frac{\alpha_{ij}^{\left(k\right)}\beta_{j}^{\left(k\right)}}{\sum_{s=1}^{M}\alpha_{is}^{\left(k\right)}\beta_{s}^{\left(k\right)}}\log\left(\alpha_{ij}\right)+\sum_{i=1}^{N}\sum_{j=1}^{M}y_{i}\frac{\alpha_{ij}^{\left(k\right)}\beta_{j}^{\left(k\right)}}{\sum_{s=1}^{M}\alpha_{is}^{\left(k\right)}\beta_{s}^{\left(k\right)}}\log\left(\beta_{ij}\right)\;=Q\left(\alpha |\theta^{\left(k\right)}\right)+Q\left(\beta |\theta^{\left(k\right)}\right).$$

#### M step

In this step, we seek the maximizer of *Q*(*θ|θ* ^(^*^k^*^)^), that is, find ^θ(k+1) =^ argmax *Q*(*θ|θ*^(^*^k^*^)^).

Denote $\Phi_{ij}=y_{i}\frac{\alpha_{ij}^{\left(k\right)}\beta_{j}^{\left(k\right)}}{\sum_{s=1}^{M}\alpha_{is}^{\left(k\right)}\beta_{s}^{\left(k\right)}}$, then we have $Q\left(\alpha |\theta^{\left(k\right)}\right)=\sum_{j=1}^{M}\sum_{i=1}^{N}\Phi_{ij}\log\left(\alpha_{ij}\right)$.

Using the Lagrangian method to maximize this function with respect to *α_ij_*s, subject to the constraint $\sum_{i=1}^{N}\alpha_{ij}=1$, *j* = 1, 2,…, *M*, we obtain the unique stationary point:

$$\alpha_{ij}=\frac{\Phi_{ij}}{\sum_{i=1}^{N}\Phi_{ij}}=\frac{y_{i}\frac{\alpha_{ij}^{\left(k\right)}\beta_{j}^{\left(k\right)}}{\sum_{s=1}^{M}\alpha_{is}^{\left(k\right)}\beta_{s}^{\left(k\right)}}}{\sum_{i=1}^{N}y_{i}\frac{\alpha_{ij}^{\left(k\right)}\beta_{j}^{\left(k\right)}}{\sum_{s=1}^{M}\alpha_{is}^{\left(k\right)}\beta_{s}^{\left(k\right)}}},$$

where *i =*1*,*…*, N, j =*1,… *M*.

Similarly, for *Q*(*β|θ*^(^*^k^*^)^), we have

$$Q\left(\beta |\theta^{\left(k\right)}\right)=\sum_{j=1}^{M}\left(\sum_{i=1}^{N}y_{i}\frac{\alpha_{ij}^{\left(k\right)}\beta_{j}^{\left(k\right)}}{\sum_{s=1}^{M}\alpha_{is}^{\left(k\right)}\beta_{s}^{\left(k\right)}}\right)\log\left(\beta_{j}\right)=\sum_{j=1}^{M}\Psi_{j}\log\left(\beta_{j}\right)$$

where $\Psi_{j}=\sum_{i=1}^{N}\Phi_{ij}$. Maximizing *Q*(*β|θ*^(^*^k^*^)^) with respect to *β_j_*s, subject to the constraint $\sum_{j=1}^{M}\beta_{j}=1$, we obtain the unique stationary point:

$$\beta =\frac{\Psi_{j}}{\sum_{j=1}^{M}\Psi_{j}}=\frac{\sum_{i=1}^{N}y_{i}\frac{\alpha_{ij}^{\left(k\right)}\beta_{j}^{\left(k\right)}}{\sum_{s=1}^{M}\alpha_{is}^{\left(k\right)}\beta_{s}^{\left(k\right)}}}{\sum_{j=1}^{M}\sum_{i=1}^{N}y_{i}\frac{\alpha_{ij}^{\left(k\right)}\beta_{j}^{\left(k\right)}}{\sum_{s=1}^{M}\alpha_{is}^{\left(k\right)}\beta_{s}^{\left(k\right)}}}=\frac{\sum_{i=1}^{N}y_{i}\frac{\alpha_{ij}^{\left(k\right)}\beta_{j}^{\left(k\right)}}{\sum_{s=1}^{M}\alpha_{is}^{\left(k\right)}\beta_{s}^{\left(k\right)}}}{\sum_{i=1}^{N}y_{i}},$$

where *j*=1,…, *M*. For any parameter, the iteration continues until the absolute change of two consecutive estimates is less than 10^−6^.

After the convergence of E-M iterations, PLSA modeling constructs the below decomposition of the vector of observed read counts, by introducing the latent variables:

$$(\begin{array}{l} y_{1}\\ y_{2}\\ \vdots \\ y_{N} \end{array})≅\left(\sum_{i=1}^{N}y_{i}\right)\left(\begin{array}{llll} \alpha_{11} & \alpha_{12} & \cdots & \alpha_{1M}\\ \alpha_{21} & \alpha_{22} & \cdots & \alpha_{2M}\\ \vdots & \vdots & \ddots & \vdots \\ \alpha_{N1} & \alpha_{N2} & \cdots & \alpha_{NM} \end{array}\right)\left(\begin{array}{l} \beta_{1}\\ \beta_{2}\\ \vdots \\ \beta_{M} \end{array}\right)$$

The approximation symbol is used to reflect the fact that the left hand side is a vector of integers (counts), while the decomposition in the right hand side may result in non-integer output. The MLEs of *β_j_*’s will then serve as the estimate of the proportions of the estimated existing COG families. Then, the estimated read count to COG *c_j_* can be computed as

$${\hat{y}}_{j}=\left[\left(\sum_{i=1}^{N}y_{i}\right){\hat{\beta }}_{j}\right],$$

where [ ] denotes the round function and *β̂_j_* is the MLE of *β_j_, j*= 1,2,…, *M.* We implemented PLSA modeling in R (http://www.r-project.org). An R script is available upon request.

### Statistical learning about the initial values for PLSA modeling

Generally, the result by iterative MLE approach is sensitive to the initial values, since the algorithm may reach the local maximization. Two assumptions have been made in order to learn the initial values of parameters. First we assume that, for the reads originating from the CDSs associated to a common COG, the frequencies of reads assigned to different COGs are similar across samples. Second, for the reads aligned to a common COG, the frequencies of the reads originating from CDSs associated to different COGs, which appear in considered samples, do not change dramatically either. Thus, we can learn the distributions from one simulated metagenomic sample, and then use the learned distribution to set the initial values for PLSA modeling for another simulated or real sample.

The learned $\alpha_{ij}^{L}$ was computed as the percentage of the reads being aligned to COG a_i_ among the reads originating from COG *c_j_*, in the learning sample. Let *γ_ji_* be the conditional probability of a read originating from *c_j_* given it being aligned to *a_i_*. Empirically, the learned $\gamma_{ji}^{L}$ was calculated as the observed relative frequency of reads originating from *c_j_* in all the reads assigned to *a_i_*. In the following, we describe in detail how to set the initial values for PLSA modeling in a sample different from the learning sample.

#### The initial value of α

1. For an estimated existing COG family c_j_, which is also present in the learning sample, we directly take $\alpha_{ji}^{L}$ as the initial value if the corresponding aligned COG appears in both samples. Otherwise, if the corresponding aligned COG appears in the new sample only, the initial value is set as the ratio of the remaining probability, $1-\sum_{i\in \cap }\alpha_{ij}^{L}$, and the number of the aligned COGs that appear in the new sample only, where *i* ∈ ∩ means that the summation is over all the aligned COGs appearing in both samples.
2. For an estimated existing COG family shown in the new sample but not in the learning sample, we set the equal initial value as probability $\frac{1}{N}$ for each aligned COG.

#### The initial value of β

1. For an estimated existing COG family *c_j_*, which is also in the learning sample, the initial value for $\beta_{j}^{L}$ is set as,
  $$\beta_{j}^{L}=\sum_{i\in \cap }\gamma_{ji}^{L}\frac{y_{i}}{\sum_{l=1}^{N}y_{l}},$$
  where *i* ∈ ∩ has the same meaning as above.
2. For the estimated existing COGs shown in the new sample only, they share the same initial value, that is, the ratio of the remaining probability, $1-\sum_{j\in \cap }\beta_{j}^{L}$, and the number of the estimated existing COGs appearing in the new sample only.

## Results

### Results from the *in vitro*-simulated metagenomic data set

We used the simulated metagenomic datasets M2 and M3 in [25], which are 4X read-coverage data, and named M2_4X and M3_4X here. In the simulations, sequencing reads with about 100 bases were produced for different preselected bacterial genomes by 454 GS20 platforms. The description about these genomes is given in Supplementary file. These data were generated through a genuine sequencing process; therefore they can best capture the characteristics of the sequencing errors introduced by 454 GS20 platforms. The related genome references were downloaded from NCBI website, with the files that contain the locations of COG coding sequences (COG-CDS) on the genomes. BLAST (that is, blastn) was applied to align the short reads against the references. The best-hit alignment with identical match greater than 95% determined where a read comes from (genome, location), otherwise the read was excluded. If the location of a read overlaps with the coverage of a COG-CDS by at least 60 bases, we consider this COG as the correct annotation for the read.

**Figure 2:**
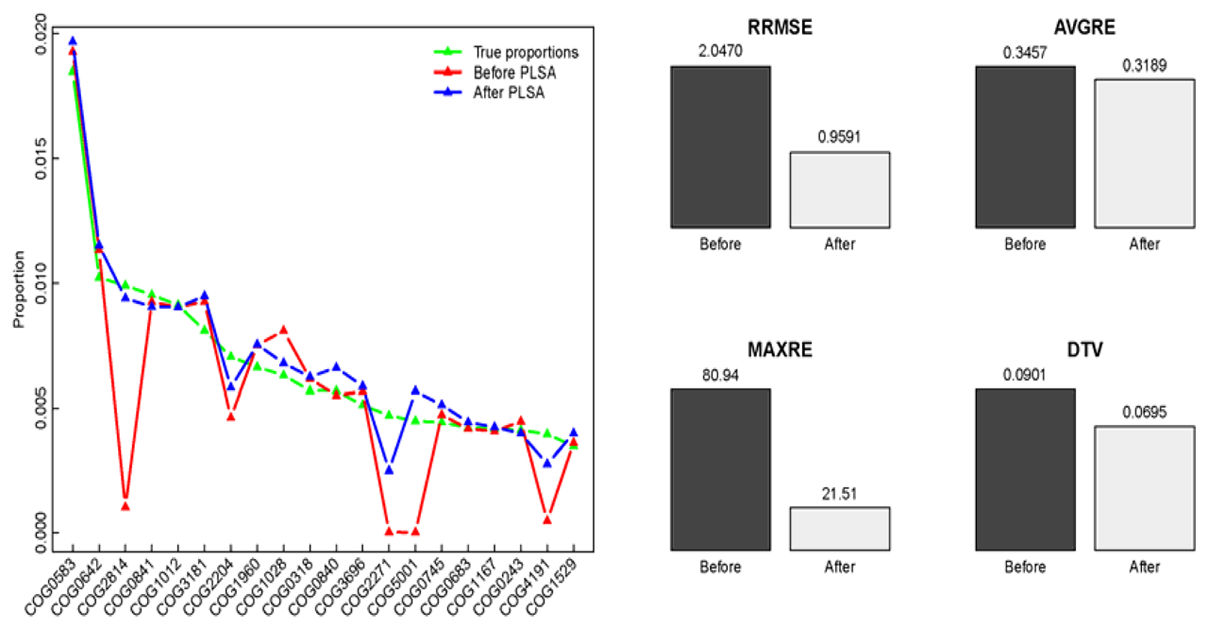
Comparison of COG functional profiles of M2_4X before and after the cross-annotation corrected by PLSA modeling: the estimated proportions of the truly most 20 abundant COGs (left); the accuracies of the estimates of the complete functional profiles, evaluated by the four measurements (right).

Following the steps given in Section 2.1, M3_4X were BLAST aligned against the COG database, and the output alignments with similarity score greater than 66 were kept to serve as a learning sample. M2_4X is the data set we used to evaluate the proposed methods. In Figure 2 we compare the propositions of COG families generated with (“After PLSA” in the plot) and without (“Before PLSA” in the plot) our proposed method to the true propositions. Note, since here we address the cross-annotations within the filtered BLAST result, the true proportions in the plot were generated by the reads with similarity scores above 66 only. The left panel presents the propositions of the 20 most abundant truly existing COGs; while the right panel lists the accuracies of the estimates of the complete functional profiles, evaluated by four measurements:

$$\text{the}\;\text{root}\;\text{relative}\;\text{mean}\;\text{square}\;\text{error}\;(\text{RRMSE}):\sqrt{\frac{1}{M-1}\sum_{j=1}^{M-1}{\left(\frac{{\hat{\beta }}_{j}-\beta_{j}}{\beta_{j}}\right)}^{2}},$$

$$\text{the}\;\text{average}\;\text{relative}\;\text{error}\;(\text{AVGRE}):\frac{1}{M-1}\sum_{j=1}^{M-1}\frac{|{\hat{\beta }}_{j}-\beta j|}{\beta j},$$

$$\text{the}\;\text{maximum}\;\text{relative}\;\text{error}\;(\text{MAXRE}):\max_{j}\left\{\frac{|{\hat{\beta }}_{j}-\beta_{j}|}{\beta_{j}}\right\},\text{and}$$

$\text{the}\;\text{total}\;\text{variation}\;\text{distance}\;(\text{DTV}):\frac{1}{2}\sum_{j=1}^{M-1}|{\hat{\beta }}_{j}-\beta_{j}|$ [26–28]. For each of the four measurements, the lower the value, the more accurate the method is. Both panels in Figure 2 indicate that the accuracy of the functional profiling of M2_4X is further improved by applying the PLSA method.

**Figure 3:**
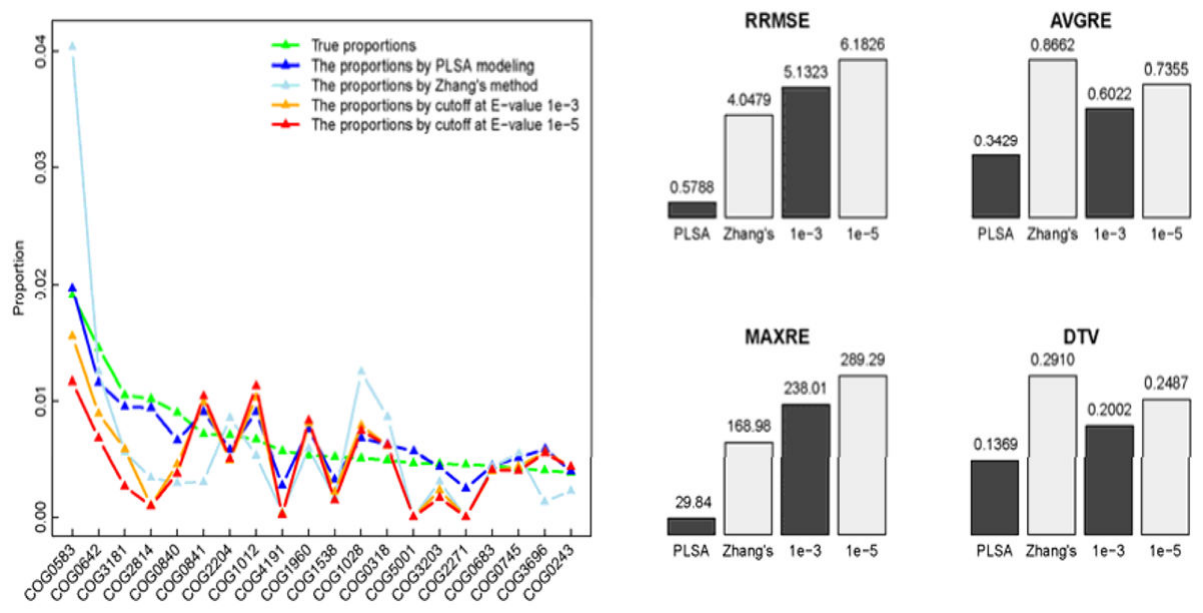
Comparison of COG functional profiles of M2_4 generated by the different methods: the estimated proportions of the truly most 20 abundant COGs (left); the accuracies of the estimates of the complete functional profiles, evaluated by the four measurements (right).

We also compared the functional profiles, in Figure 3, generated separately by our PLSA modeling method, the method proposed in Zhang et al., [16], and the two currently used E-value cutoff methods (1e-3, 1e-5). Note, in order to compare different methods, the true proportions are generated using all the sequencing reads with detected genomic locations. Due to the facts that Zhang’s method was summarized from a single simulation, and that the problems of both artificial families and cross-annotations are ignored by the E-value cutoff methods, the abundance proportions of certain COGs are skewed greatly in the profiles generated by these methods. In the true profile, COG2814 is ranked the fourth abundant family; however, it is ranked the 27th in Zhang’s method, the 196th and 243th abundant in the profiles by E-value cutoff at 1e-3 and 1e-5 respectively. In the profile by our method, this functional family is correctly annotated as the fourth abundant one. Similar situations can be observed for the families: COG4191, COG5001 and COG2271 (left panel in Figure 3). For example, COG5001 is ranked the 14^th^, 13^th^, 2246^th^, 2230^th^ and 2215^th^ abundant respectively in the true profile, the profiles by our method, Zhang’s method, E-value cutoff at 1e-3 and 1e-5. Its actual proportion of 0.0047 is closely estimated as 0.0056 by our method; but the estimation drops dramatically to 3.9e-6, 6.6e-6 and 4.6e-6 respectively in the other three profiles, erroneously indicating that the family is very trivial. On the other hand, we observed that certain trivially abundant entries in the true profile, such as COG0784 (1866^th^), COG0067 (2024^th^) and COG0506 (2255^th^), become non-trivial in profiles by Zhang’s method and the methods of E-value cutoff (not appear in the plot). As an example, COG0784 becomes non-trivial (ranks the 77^th^, 27^th^ and 70^th^ respectively) in the profiles by the three methods. Evaluated by the above four measurements, the estimate of the complete functional profile by our PLSA method also shows the best accuracy (the lowest bar in the right panel in Figure 3).

### Results from metagenomic data set simulated by a bioinformatics tool

**Figure 4:**
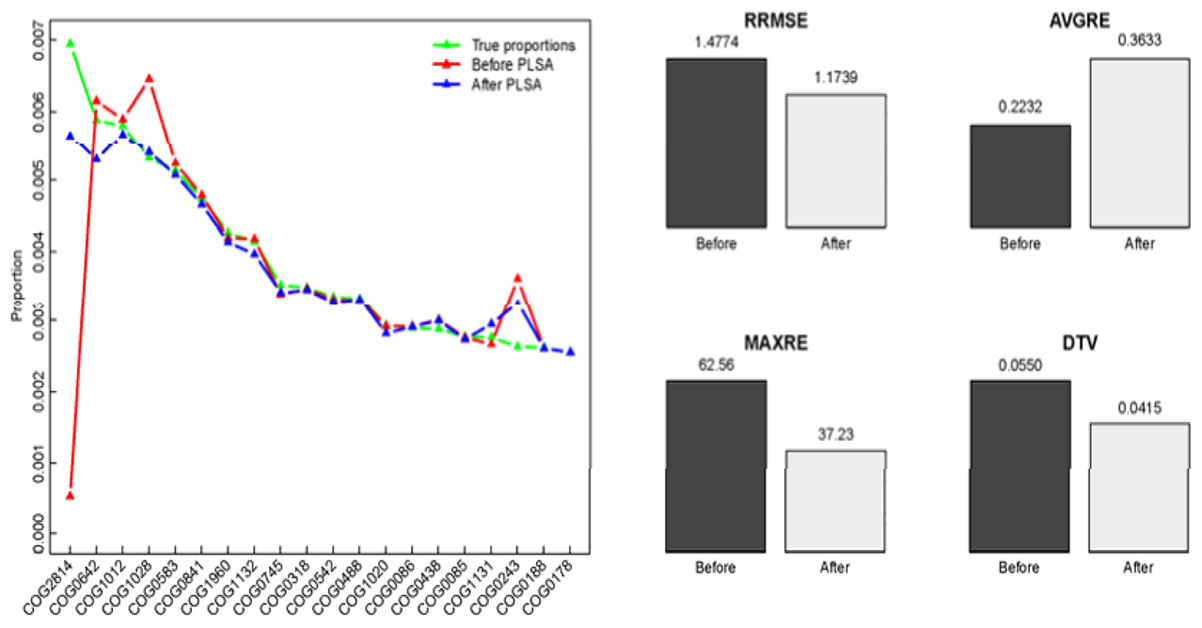
Comparison of COG functional profiles of Simu before and after the cross-annotation corrected by PLSA modeling method: the estimated proportions of the truly most 20 abundant COGs (left); the accuracies of the estimates of the complete functional profiles, evaluated by the four measurements (right).

The numbers of species in the samples in Section 5.1 are 7 (M2_4x) and 8 (M3_4X), usually smaller compared to the real-life metagenomic data. Thus, in this subsection, we would evaluate the proposed method on a metagenomic sample with large species diversity. We randomly selected 100 NCBI bacterial genome accession numbers (in the format of NC_######), among which 57 genomes were excluded in the simulation since they were for plasmid DNA sequences. MetaSim, a bioinformatic tool to simulate sequencing reads according to selected genomes, was used to generate the metagenomic data set, called Simu. The data set contains the short reads of 43 genomes with coverage one under the simulated conditions of 454 GS20 sequencing platform (see a brief description about these genomes, and the parameters used for simulation in Supplementary File). Since we know exactly where a read originates from, we did not use the blastn step as we did in Section 5.1. As before, the correct annotation of a read is defined as the COG whose coverage overlaps with the location of the read by at least 60 bases. To apply our method for modelling this simulated data set, we selected the learning data as the combination, called M_4X (available upon request), of all the three 4X read-coverage data sets of the simulated metagenomes M1, M2 and M3 in [25]. The reason we combined these 4X read-coverage data as the learning set is that this would provide us with more observed reads originating from a common COG, thus we will have a better statistical learning result about the distribution of these reads being aligned among COGs. On the other hand, with more reads being aligned to a common COG, we would have a better learning about the distribution of reads originating from COGs as well. The results presented in Figure 4 exhibit the profiles of COG families generated before and after applying the proposed PLSA modeling method. The finding is similar to those from Figure 2 in that the accuracy of the functional profiling can be improved by the proposed method, except the AVGRE measurement. A partial explanation for this is that the proposed method smooth the relative errors.

**Figure 5:**
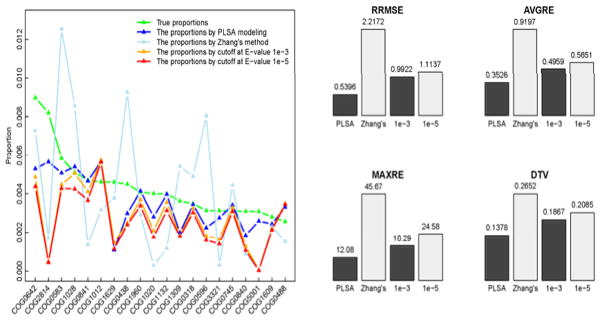
Comparison of COG functional profiles of Simu generated by different methods: the estimated proportions of the truly most 20 abundant COGs (left); the accuracies of the estimates of the complete functional profiles, evaluated by the four measurements (right).

The comparison between PLSA modeling approach and the other three methods using data set Simu is presented in Figure 5. It is clear that (the left panel) the proportion of COG2814 is poorly estimated by the three methods (the true proportion: 0.0082; the proportion by Zhang’s method: 0.0017; the proportion by cutoff 1e-3: 0.00048; the proportion by the cutoff 1e-5: 0.00045). The estimation is greatly improved to 0.0057 by our method. As a trivial abundant family, COG0784 has the true proportion of 1.9×10^−5^, which is estimated as 3.07×10^−5^, by our method (not appear in the plot). Its abundance is greatly inflated to a significant entry in the profiles generated by the two E-value cutoff methods (the cutoff 1e-3: 0.0013; the cutoff 1e-5: 0.0011). For the estimate of the complete functional profile, PLSA modeling method provides the best accuracy giving the lowest error in each of four measurements (the right panel in Figure 5).

### Application of the proposed method on a real data set

**Figure 6:**
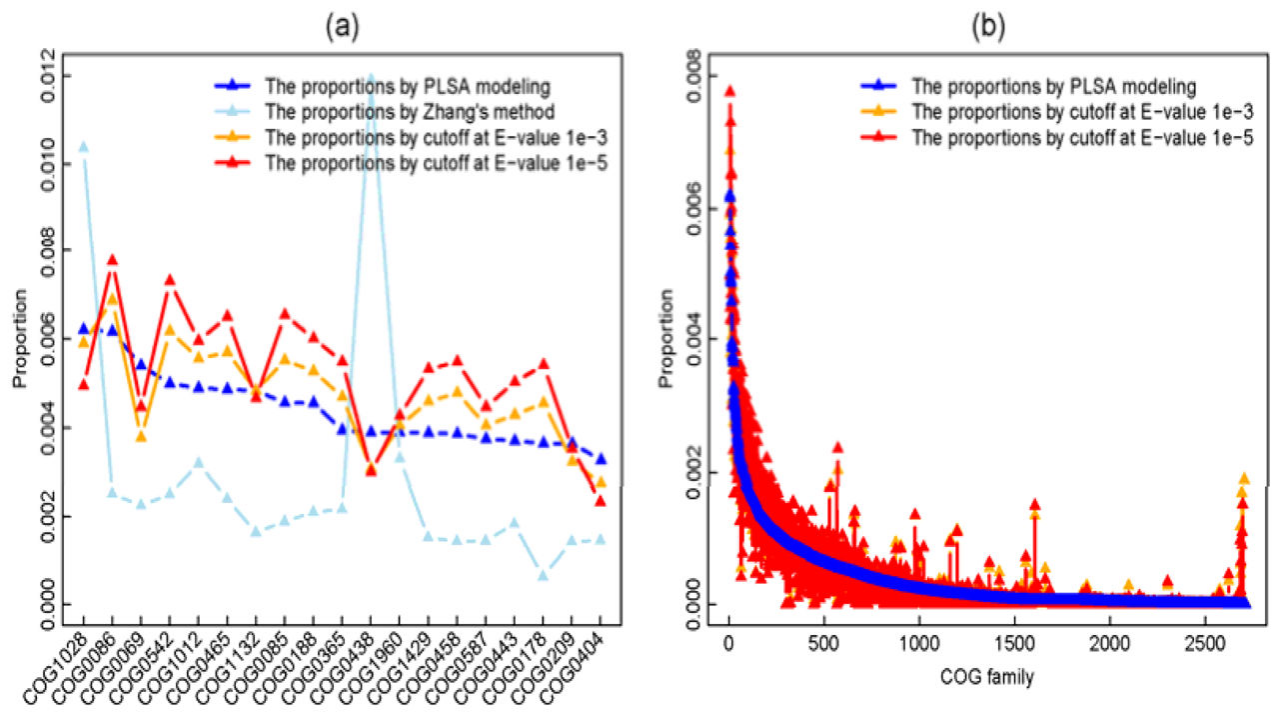
Comparison of COG functional profiles of HOT25: (a) the estimated proportions of the truly most 20 abundant COGs; (b) the complete functional profiles generated by PLSA modeling, E-value cutoff at 1e-3 and 1e-5.

A picoplanktonic sample was collected from 25m-depth seawater at the Hawaii Ocean Time Series (HOT) station on March, 2006. It was then sequenced with 454 GS20 machine to yield 385,193 short reads of 108 bases long on average [2]. We name this data set as HOT25. By using M_4X as the learning data, we applied he proposed PLSA modeling approach to correct the cross-annotations in its BLAST output (the one after filtration with similarity score cutoff 66). For the top 20 most abundant COGs estimated by PLSA modeling method, Figure 6a shows the discrepancy of the abundances given by the four approaches (the PLSA modeling method, Zhang’s method, E-value Cutoffs 1e-3 and 1e-5). Unlike the simulated data, prior information on COG families for real data are not available, and thus, cannot be used to show the closeness of these profiles to the true one. The comparison of the complete functional profiles is displayed in Figure 6b, with the profile generated by Zhang’s method being excluded since it is too different to compare. We can see that some COGs are estimated as very trivial ones by PLSA modeling method, but significant ones by E-value cutoff methods (the red/orange triangles close to the right tail of the blue curve).

Table 1 lists three functional families, COG1028, COG0642 and COG0477, with corresponding proportions estimated by the methods and the ranks of abundances in each generated profile. A recent study has detected COG1028 is the most abundant COG family in the metagenomic samples from HOT station, the second abundant in the samples collected from western Arctic Ocean, and the third in the samples from the coastal water near Cape May, NJ [29,30]. The COG1028 belongs to the COG category I, Lipid transport and metabolism, demonstrating the universally important roles in different latitude of seawater. The PLSA modeling method also ranks COG1028 as the most abundant one in the HOT25 sample, but the other methods do not reach to the same conclusion. The family COG0642 comes from COG category T, Signal transduction. This mechanism is important for microbes to cope with changing environmental conditions. The role of COG0642 has been examined in many seawater related metagnomic projects; its abundance level is found varied in different depth of water since the environmental stimuli, such as temperature and sunlight, are different [31–34]. For the HOT25 sample, the COG0642 is estimated as the 51^st^ abundant family by the PLSA modeling method, but ranked very differently, 86th and 131th separately, by the E-value cutoff methods. There are also some research records about COG0477. In order to understand how bacterioplankton transform dissolved organic carbon in marine systems, Mou [35] conducted metagenomic analysis of bacterioplankton enriched with dimethylsulfoniopropionate (DMSP) and vanillic acid (VanA). Sequencing reads with an average length of 97 bases were obtained by pyrosequencing. The reads were aligned to COG database, and the abundance of each COG family was obtained. Furthermore, PCR-based 16S rDNA analysis was also carried out in the same project. For COG0477, its abundances in both DMSP and VanA samples were found very high by the metagenomic approach; but, interestingly there are no genes associated to COG0477 in the genomes detected by 16SrDNA analysis using the same samples. This is another supporting evidence about artifacts and cross-annotations when short reads being annotated. Note that different from E-value cutoff methods, both PLSA modeling method and Zhang’s method give zero abundance to this family.

## Discussion

Due to the fact that a microbial community usually includes multiple strains or similar species, the algorithms for assembling sequencing reads generated from a single genome are not applicable to metagenomic reads. On the other hand, accurate assembly really depends on sufficient sequencing depth [36], while a metagenomic sample generally consists of data with lower sequencing depth. Prior to 2013, the development of metagenomic assembly was evaluated as “in its infancy” [37,38], “at an early stage” [36]. And, to our knowledge, since 2013, there has been no breakthrough that can provide a widely accepted assembling tool. Therefore, we chose to analyze the unassembled short reads directly in this study. There might be an argument that assembled reads would reduce the cross-annotations. However, as a recent study indicated, there is a price for using assembled reads – it can bring in a considerable proportion of chimeric contigs [39], which is even harder to deal with in our opinion. Analysis of unassembled metagenomic reads is one of the approaches currently employed to study microbial communities [40–43]. Our method can be adapted to handle short reads with different lengths (e.g. ~200 bases), given a good alignment cutoff value and a trustable learning data set. We conducted the study specifically on reads with ~100 bases owing to two reasons: first, the alignment cutoff value for reads with ~100 bases has been suggested [23], and PLSA modeling method was applied on the filtered result; second, through literature search, the *in vitro-*simulated metagenomic samples are only available with this length range. Should similar samples with other lengths become available, we will enlarge the application scope of the method.

In addition to the issues of artificial COGs and cross-annotations, it has been reported in the literature that another problem exists with read count bias in metagenomic data [16]. Briefly speaking, the count of reads aligned to a COG family is correlated with the lengths and the conservations of COG-CDSs associated to the COG. This bias may have impact on the accuracy of the functional profile of COG families and deserves further investigation. To study and correct the read bias is one of our future research topics.

## Supplementary Material

Supplemental Data

## Figures and Tables

**Table 1:** Proportions and ranks of three COGs by different methods.

	PLSA		Zhang’s		E 1e-3		E 1e-5	
	prop.	rank	prop.	rank	prop.	rank	prop.	rank
COG1028: Dehydrogenases with different specificities	0.0062	1	0.01	3	0.0059	3	0.0049	13
COG0642: Signal transduction histidine kinase	0.0022	51	0.0031	22	0.002	86	0.0018	131
COG0477: Permeases of the major facilitator superfamily	0	NA	0	NA	0.0015	161	0.0011	276

## References

[1] Handelsman J (2004). Metagenomics: application of genomics to uncultured microorganisms. Microbiol Mol Biol Rev.

[2] Frias-Lopez J, Shi Y, Tyson GW, Coleman ML, Schuster SC (2008). Microbial community gene expression in ocean surface waters. Proc Natl Acad Sci USA.

[3] Rajendhran J, Gunasekaran P (2008). Strategies for accessing soil metagenome for desired applications. Biotechnol Adv.

[4] Edwards RA, Rodriguez-Brito B, Wegley L, Haynes M, Breitbart M (2006). Using pyrosequencing to shed light on deep mine microbial ecology. BMC Genomics.

[5] Lazarevic V, Whiteson K, Huse S, Hernandez D, Farinelli L (2009). Metagenomic study of the oral microbiota by Illumina high-throughput sequencing. J Microbiol Methods.

[6] Sturgeon A, Stull JW, Costa MC, Weese JS (2013). Metagenomic analysis of the canine oral cavity as revealed by high-throughput pyrosequencing of the 16S rRNA gene. Vet Microbiol.

[7] Turnbaugh PJ, Ley RE, Mahowald MA, Magrini V, Mardis ER (2006). An obesity-associated gut microbiome with increased capacity for energy harvest. Nature.

[8] Qin J, Li R, Raes J, Arumugam M, Burgdorf KS (2010). A human gut microbial gene catalogue established by metagenomic sequencing. Nature.

[9] Dinsdale EA, Edwards RA, Hall D, Angly F, Breitbart M (2008). Functional metagenomic profiling of nine biomes. Nature.

[10] Harrington ED, Singh AH, Doerks T, Letunic I, von Mering C (2007). Quantitative assessment of protein function prediction from metagenomics shotgun sequences. Proc Natl Acad Sci USA.

[11] Tatusov RL, Galperin MY, Natale DA, Koonin EV (2000). The COG database: a tool for genome-scale analysis of protein functions and evolution. Nucleic Acids Res.

[12] Tatusov RL, Fedorova ND, Jackson JD, Jacobs AR, Kiryutin B (2003). The COG database: an updated version includes eukaryotes. BMC Bioinformatics.

[13] Koonin EV, Fedorova ND, Jackson JD, Jacobs AR, Krylov DM (2004). A comprehensive evolutionary classification of proteins encoded in complete eukaryotic genomes. Genome Biol.

[14] Finn RD, Tate J, Mistry J, Coggill PC, Sammut SJ (2008). The Pfam protein families database. Nucleic Acids Res.

[15] Haft DH, Selengut JD, White O (2003). The TIGRFAMs database of protein families. Nucleic Acids Res.

[16] Zhang Q, Doak TG, Ye Y (2012). Artificial functional difference between microbial communities caused by length difference of sequencing reads. Pac Symp Biocomput.

[17] Mardis ER (2008). The impact of next-generation sequencing technology on genetics. Trends Genet.

[18] Xu L, Chen H, Hu X, Zhang R, Zhang Z (2006). Average gene length is highly conserved in prokaryotes and eukaryotes and diverges only between the two kingdoms. Mol Biol Evol.

[19] Altschul SF, Gish W, Miller W, Myers EW, Lipman DJ (1990). Basic local alignment search tool. J Mol Biol.

[20] Altschul SF, Madden TL, Schäffer AA, Zhang J, Zhang Z (1997). Gapped BLAST and PSI-BLAST: a new generation of protein database search programs. Nucleic Acids Res.

[21] Altschul SF, Gish W (1996). Local alignment statistics. Methods Enzymol.

[22] Wommack KE, Bhavsar J, Ravel J (2008). Metagenomics: read length matters. Appl Environ Microbiol.

[23] Du R, Mercante D, Fang Z (2013). An artificial functional family filter in homolog searching in next-generation sequencing metagenomics. PLoS One.

[24] Hofmann T (1999). Probabilistic latent semantic indexing.

[25] Byrd A, Perez-Rogers JF, Manimaran S, Castro-Nallar E, Toma I (2014). Clinical PathoScope: rapid alignment and filtration for accurate pathogen identification in clinical samples using unassembled sequencing data. BMC bioinformatics.

[26] Burke C, Steinberg P, Rusch D, Kjelleberg S, Thomas T (2011). Bacterial community assembly based on functional genes rather than species. Proc Natl Acad Sci USA.

[27] Cottrell MT, Kirchman DL (2012). Virus genes in Arctic marine bacteria identified by metagenomic analysis. Aquatic Microbial Ecology.

[28] Dalevi D, Ivanova NN, Mavromatis K, Hooper SD, Szeto E (2008). Annotation of metagenome short reads using proxygenes. Bioinformatics.

[29] DeLong EF, Preston CM, Mincer T, Rich V, Hallam SJ (2006). Community genomics among stratified microbial assemblages in the ocean’s interior. Science.

[30] Eloe EA, Fadrosh DW, Novotny M, Zeigler Allen L, Kim M (2011). Going deeper: metagenome of a hadopelagic microbial community. PLoS One.

[31] White NA, Engeman RM, Sugihara RT, Krupa HW (2008). A comparison of plotless density estimators using Monte Carlo simulation on totally enumerated field data sets. BMC Ecol.

[32] Francis OE, Bendall M, Manimaran S, Hong C, Clement NL (2013). Pathoscope: species identification and strain attribution with unassembled sequencing data. Genome Res.

[33] Liu JS (2008). Monte Carlo strategies in scientific computing.

[34] Mou X (2006). Culture-independent characterization of DOC-transforming bacteriplankton in coastal seawater.

[35] Nagarajan N, Pop M (2013). Sequence assembly demystified. Nat Rev Genet.

[36] Richter DC, Ott F, Auch AF, Schmid R, Huson DH (2008). MetaSim: a sequencing simulator for genomics and metagenomics. PLoS One.

[37] Singh AH, Doerks T, Letunic I, Raes J, Bork P (2009). Discovering functional novelty in metagenomes: examples from light-mediated processes. J Bacteriol.

[38] Skennerton CT, Imelfort M, Tyson GW (2013). Crass: identification and reconstruction of CRISPR from unassembled metagenomic data. Nucleic Acids Res.

[39] Thomas T, Gilbert J, Meyer F (2012). Metagenomics-a guide from sampling to data analysis. Microb Inform Exp.

[40] Vázquez-Castellanos JF, García-López R, Pérez-Brocal V, Pignatelli M, Moya A1 (2014). Comparison of different assembly and annotation tools on analysis of simulated viral metagenomic communities in the gut. BMC Genomics.

[41] Wooley JC, Ye Y (2009). Metagenomics: Facts and Artifacts, and Computational Challenges. J Comput Sci Technol.

[42] Wu J, Gao W, Johnson RH, Zhang W, Meldrum DR (2013). Integrated metagenomic and metatranscriptomic analyses of microbial communities in the meso- and bathypelagic realm of north pacific ocean. Mar Drugs.

[43] Xia LC, Cram JA, Chen T, Fuhrman JA, Sun F (2011). Accurate genome relative abundance estimation based on shotgun metagenomic reads. PLoS One.

