# Supplementary material for: A Statistical Approach to Correcting Cross-Annotations in a Metagenomic Functional Profile Generated by Short Reads
Source: J Biom Biostat. Author manuscript; Available in PMC 2018 Apr 27. (PMC5922784; doi:10.4172/2155-6180.1000208)
Supplement: Supplemental Data [file NIHMS949870-supplement-Supplemental_Data.pdf]

Supplementary Table 1. The list of genomes for M2.

Genome Accession #	Organism Name	Genome Length	NO. Reads Simulated			
			01X	1X	2X	4X
NC_011979	*Geobacter sp*. FRC-32	3 982463	4225	42266	84525	158487
NC_010084<br>NC_010086<br>NC_010087	*Burkholderia multivorans* ATCC 17616	6 979389	7110	71074	142102	284221
NC_010002	M2 *Delftia acidovorans* SPH-1	6 702581	7046	70448	140916	267735
NC_013446	*Comamonas testosteroni* KF-1	5 906374	6189	61895	123794	237264
NC_010814	*Geobacter lovleyi* SZ	3 871860	4300	43004	86009	153584

Supplementary Table 2. The list of genomes for M3.

Genome Accession #	Organism Name	Genome Length	NO. Reads Simulated			
			01X	1X	2X	4X
NC_009438	*Shewanella putrefaciens* CN-32	4 659220	4714	47151	94318	188633
NC_009092	*Shewanella loihica* PV-4	4 602594	4588	45882	91773	183536
NC_008789	*Halorhodospira halophila* SL1	2 678452	2690	26898	53796	110282
NC_009512	M3 *Pseudomonas putida* F1	5 959964	6407	64080	128158	238005
NC_009997	*Shewanella baltica* OS195	5 310173	5378	53779	107548	215103
NC_011593	*Bifidobacterium longum* bv. Infantis ATCC 15697	2 832748	2898	28990	57981	112343
NC_011071	*Stenotrophomonas maltophilia* R551-3	4 544233	4685	46844	93699	179581
NC_009719	*Parvibaculum lavamentivorans* DS-1	3 854587	4501	39379	78764	157526

Supplementary Table 3. The list of genomes in the simulated data set *Simu*.

Genome Accession #	Organism Name	Genome Length (bases)	NO. Reads Simulated
NC_011312	*Aliivibrio salmonicida LFI1238*	3325165	33103
NC_009922	*Alkaliphilus oremlandii OhILAs*	3123558	31500
NC_007716	*Aster yellows witches'-broom phytoplasma AYWB*	706569	6973
NC_014829	*Bacillus cellulosilyticus DSM 2522*	4681672	46702
NC_014019	*Bacillus megaterium QM B1551*	5097129	51138
NC_013791	*Bacillus pseudofirmus OF4*	3858997	38354
NC_010581	*Beijerinckia indica subsp. indica ATCC 9039*	4170153	41466
NC_014616	*Bifidobacterium bifidum S17*	2186882	21732
NC_011229	*Borrelia duttonii Ly*	931674	9266
NC_008513	*Buchnera aphidicola str. Cc (Cinara cedri)*	416380	4196
NC_010280	*Chlamydia trachomatis L2b/UCH-1/proctitis*	1038863	10508
NC_002491	*Chlamydophila pneumoniae J138*	1226565	12212
NC_010723	*Clostridium botulinum E3 str. Alaska E43*	3659644	36555
NC_015856	*Collimonas fungivorans Ter331*	5186898	52002
NC_015185	*Desulfurobacterium thermolithotrophum DSM 11699*	1541968	15552
NC_013037	*Dyadobacter fermentans DSM 18053*	6967790	69332
NC_014121	*Enterobacter cloacae subsp. cloacae ATCC 13047*	5314581	53292
NC_015601	*Erysipelothrix rhusiopathiae str. Fujisawa*	1787941	17670
NC_015672	*Flexistipes sinusarabici DSM 4947*	2526590	25147
NC_014394	*Gallionella capsiferriformans ES-2*	3162471	31424
NC_014366	*Gamma proteobacterium HdN1*	4587455	45962
NC_005125	*Gloeobacter violaceus PCC 7421*	4659019	46677
NC_010125	*Gluconacetobacter diazotrophicus PAl 5*	3944163	39491
NC_011283	*Klebsiella pneumoniae 342*	5641239	56361
NC_004343	*Leptospira interrogansserovar Lai str. 56601*	359372	3634
NC_015516	*Melissococcus plutonius ATCC 35311*	1891014	18857
NC_007355	*Methanosarcina barkeri str. Fusaro*	4837408	48186
NC_010172	*Methylobacterium extorquens PA1*	5471154	54893
NC_002977	*Methylococcuscapsulatus str. Bath*	3304561	32742
NC_008596	*Mycobacterium smegmatis str. MC2 155*	6988209	70227
NC_004757	*Nitrosomonas europaea ATCC 19718*	2812094	28214
NC_012440	*Persephonella marina EX-H1*	1930284	19434
NC_011147	*Salmonella enterica subsp. Enterica serovar Paratyphi A str. AKU_12601*	4581797	45792
NC_014168	*Segniliparus rotundus DSM 44985*	3157527	31654
NC_013929	*Streptomyces scabiei 87.22*	10148695	101748
NC_015953	*Streptomyces sp. SirexAA-E*	7414440	74626
NC_012622	*Sulfolobus is landicus Y.G.57.14*	2702058	26987
NC_014506	*Sulfurimonas autotrophica DSM 16294*	2153198	21471
NC_006461	*Thermus thermophilus HB8*	1849742	18229

Supplementary Information: the parameters used for generating *Simu* by MetaSim.

```
Simulator Settings:
Preset Name: 454
Number Of Reads / Mate Pairs=1974269
Error Model=454

454 Error Model Configuration=
Number Of Cycles: 39 (~99 Base Pairs)
Mate Pair Probability: 0.0
Mate Pair Read Length: 20
Remove Mate Pair Linker from Output: true
Lognormal Distribution Mean: 0.23
Lognormal Distribution Std. Deviation: 0.15
Proportionality Constant for Std. Deviation: 0.15
Scale Std. Deviation with Square Root of Mean: true
Generate Signal Trace: false

454 Error Model DNA Clone Parameters=
Distribution: Normal
Mean: 2000.0
2nd parameter: 200.0

Combine All Files=false
Uniform Sequence Weights=false
Number Of Threads=1
Write FastA=true
Compress Output Files=false
```
